# Stability and Reproducibility of Radiomic Features Based Various Segmentation Technique on MR Images of Hepatocellular Carcinoma (HCC)

**DOI:** 10.3390/diagnostics11091573

**Published:** 2021-08-30

**Authors:** Nurin Syazwina Mohd Haniff, Muhammad Khalis Abdul Karim, Nurul Huda Osman, M Iqbal Saripan, Iza Nurzawani Che Isa, Mohammad Johari Ibahim

**Affiliations:** 1 Department of Physics, Faculty of Science, Universiti Putra Malaysia, Serdang 43400, Selangor, Malaysia; nurinsyazwina1996@gmail.com (N.S.M.H.); nurulhuda@upm.edu.my (N.H.O.); 2 Department of Computer Engineering, Faculty of Engineering, Universiti Putra Malaysia, Serdang 43400, Selangor, Malaysia; iqbal@upm.edu.my; 3 Programme of Diagnostic Imaging and Radiotherapy, Universiti Kebangsaan Malaysia, Kuala Lumpur 50300, Wilayah Persekutuan, Malaysia; zawani@ukm.edu.my; 4 Department Biochemistry & Molecular Medicine, Faculty of Medicine, Universiti Teknologi MARA, Sungai Buloh 47200, Selangor, Malaysia; mji@uitm.edu.my

**Keywords:** HCC, MRI, radiomics, manual segmentation, semi-automatic segmentation

## Abstract

Hepatocellular carcinoma (HCC) is considered as a complex liver disease and ranked as the eighth-highest mortality rate with a prevalence of 2.4% in Malaysia. Magnetic resonance imaging (MRI) has been acknowledged for its advantages, a gold technique for diagnosing HCC, and yet the false-negative diagnosis from the examinations is inevitable. In this study, 30 MR images from patients diagnosed with HCC is used to evaluate the robustness of semi-automatic segmentation using the flood fill algorithm for quantitative features extraction. The relevant features were extracted from the segmented MR images of HCC. Four types of features extraction were used for this study, which are tumour intensity, shape feature, textural feature and wavelet feature. A total of 662 radiomic features were extracted from manual and semi-automatic segmentation and compared using intra-class relation coefficient (ICC). Radiomic features extracted using semi-automatic segmentation utilized flood filling algorithm from 3D-slicer had significantly higher reproducibility (average ICC = 0.952 ± 0.009, *p* < 0.05) compared with features extracted from manual segmentation (average ICC = 0.897 ± 0.011, *p* > 0.05). Moreover, features extracted from semi-automatic segmentation were more robust compared to manual segmentation. This study shows that semi-automatic segmentation from 3D-Slicer is a better alternative to the manual segmentation, as they can produce more robust and reproducible radiomic features.

## 1. Introduction

Hepatocellular carcinoma (HCC) is of the common type of malignant tumour of the liver and its incidence has increased in recent years. In Malaysia, HCC is one of the top five cancers with 4.4% new cases in 2018, and it ranks fourth in leading causes of cancer death [1]. HCC known to have a poor survival rate despite availability varieties of clinical treatments [2]. Medical imaging techniques such as computed tomography (CT) and magnetic resonance imaging (MRI) are vital procedures in oncological treatment. Both CT scans and MRI have higher spatial resolution that has advantage in the early detection of tissues’ abnormality in patients [2]. Imaging is extensively used in detection, diagnosis, and staging of cancer. Several studies have been conducted to improve the tumour diagnosis through proposed methods [3,4,5,6]. Although MRI is acknowledged for its advantages, the false-negative diagnosis from the examinations is inevitable, although several quantitative techniques have been introduced which are mostly pertaining to MR study. It is crucial for optimizing quantitative imaging feature extraction through computational approaches and developing decision support systems to accurately estimate the cancer stage. Hence, radiomics study are introduced and used to extract high number of quantitative image features [7].

Feature extraction is essential to obtain relevant information on input images and represents data in lower dimensionality space [8]. These features are extracted by using advanced mathematical algorithm which describes phenotypes of tumour that might not be able to perceive by naked eye. Previous studies have shown the importance of quantitative feature extraction in classifying the cancer stage of various types of cancer, including HCC [9,10,11,12]. In 2016, investigation of extraction of radiomics data was performed to predict the pathological response after chemotherapy [13]. Incorporations of radiomics and machine learning are essential for information extraction to aid in better prognosis. From shape, statistics, and texture features, 20 features were selected based on stability and variance. 

Recently, uses of radiomics in cancer related field shows significant progress. Radiomics application is said to be one of the fundamental methods for machine learning development in the medical imaging field [14,15]. Extraction of radiomic features from various sources of medical images also overcomes the limitation of visual image interpretation [16]. Several literature reviews show data mining and predictive analysis have widened the scope of medical imaging [17,18,19,20]. This can facilitate prognostic models used in oncology. However, the poor reliability of radiomic features could affect research outcomes and become an obstacle for further use in models [21].

It is important to ensure the accuracy of quantitative features extracted from medical images. The process of tumour segmentation is one of the main obstacles for radiomics. Conventional manual segmentation is considered quite tedious and time consuming where quick and reliable segmentation techniques are required. Previous studies show that semi-automatic segmentation methods are preferred, as they are better alternatives to manual segmentation [22,23,24]. Comparative analysis of different types of image segmentation had been done for mammographic images in 2019. This study concludes flood filling algorithm has the best segmentation result compared to the watershed algorithm, the mean-shift algorithm, and the k-means algorithm [25]. Recently, a study was done for non-small cell lung cancer, radiomic features extracted for semi-automatic segmentation using a grow-cut algorithm implemented in 3D-Slicer, has high reproducibility and is more robust compared to manual segmentation [26]. Studies on CT and MR images prove that semi-automatic segmentation is robust, which indicates its ability to produce reliable and reproducible radiomic models [27,28]. Hence, in this study, we aim to evaluate the reproducibility and robustness of various segmentation techniques of MR HCC images based on the extract radiomic features.

## 2. Materials and Methods

In this study, we analysed robustness of features extracted from 30 patients (median age: 63 y/o) diagnosed with HCC by two different methods of segmentations, manual and semi-automatic. We extracted 662 features using 3D Slicer (Boston, MA, USA) software for both segmentations [29]. Features extractions for manual and semi-automatic segmentations were compared and analyzed. Despite having small samples size (n = 30), numbers of sets for observations were examined to find the pattern on features extracted. Briefly, a total of 240 segmentations (120 manual segmentations and 120 semi-automatic segmentations) were analyzed in this study.

### 2.1. Magnetic Resonance Imaging (MRI) of HCC Patients

The image data was obtained from open archive for medical images of cancer, The Cancer Imaging Archive (TCIA) (Arkansas, USA) [30]. Ninety-seven patients verified with hepatocellular carcinoma, Stages I–IV, were retrieved. After reviewing the data images, patients who underwent CT scans were excluded, and only non-contrast enhanced T1-weighted (T1W) images (TR/TE = 210/1.1 ms) were chosen. A total of 30 patients diagnosed using MRI were included in this study. For the final study, 17 male (median age: 64 years; range 23–77 years) and 13 females (median age: 62 years; range 45–77 years). Stages of HCC are divided into two groups, Stages I–II and Stages III–IV, respectively.

### 2.2. Semi-Automatic Segmentation in 3D-Slicer

For the semi-automatic segmentation, the flood fill algorithm from 3D Slicer software was applied on the subject MR images. Prior to the installation, the Segment Editor Extra Effects extension was installed through the extension’s manager. After loading data images through the DICOM module, the observers identify the location of HCC. The nodes were added around the tumour region using a mouse cursor. Subsequently, the flood fill effects were activated, and ROI were segmented according to similar voxels intensity. To finalize the output, the segmented tumour was manually edited in the finalization phase as semi-automatic segmentation. Flood fill is an algorithm used to determine connection of an area in multi-dimensional array with the help of similarity of intensity voxels to the selected node determined by users. This algorithm is comparable with bucket tool in paint programs, which fill connected similar intensity voxels with different colours [25]. The algorithm was initiated with a start node by selecting the region of interest (ROI). Pixels connected four-directionally with the start node and pixels connected four-directionally to the former are considered in this effect. As the intensity voxels were decided, the algorithm identified the path of target node given and replaced it with different colours. Leakage prevention to other structures under this effect were manipulated using the neighbourhood size parameter.

### 2.3. Feature Extraction

All segmented image data were analysed and performed in the same software, 3D Slicer under the Radiomics module to extract imaging features. Features were extracted using the mathematical algorithm predicated on pixel intensities. From semi-automatic and manual segmentations, we defined 662 radiomic features for MR images that assist in quantifying tumour characteristics. The features were divided into four groups: (I) tumour intensity, (II) shape, (III) texture, and (IV) wavelet-features with examples of shape features shown in Table 1. The total features extracted from volume of interest for tumour intensity, shape and textural were 18, 14, and 54 features, respectively.

Tumour intensity represents a first order statistic, which distinguishes a histogram of voxel intensity within the tumour region on MRI. Shape features are calculated and described with volume properties of the tumour. Shape features extracted using the pyRadiomics package are independent from gray-level intensities and can only be calculated on a non-derived image, which is the original image [31]. Thus, the wavelet filter is not applied to shape features as it will not be calculated on derived images. Textural features were determined by patterns or spatial distributions of voxel intensities derived from grey level dependence matrix (GLDM), grey level co-occurrence matrix (GLCM) and grey level run-length matrices (GLRLM) [32]. Features derived from co-occurrence and run-length matrices were computed by averaging all 13 symmetric directions in three dimensions [6]. 

Wavelet features are obtained by transforming domain representations of tumour intensity and textural features. These features were applied as either a high (H) or low pass (L) filter in each of the three dimensions—X-axis, Y-axis, and Z-axis: wavelet-LHL, wavelet-LHH, wavelet-HLL, wavelet-LLH, wavelet-HLH, wavelet-HHH, wavelet-HHL, and wavelet-LLL [14]. Eight decomposed volumes of images were used on the intensity and textural features in the volume of interest, which resulted in a total of 576 (8 × 72) wavelet transforms features [7,23]. These filters help to identify the detail or sudden changes in intensity in the image [33]. Several fundamental formulas, such as entropy, contrast, uniformity, and correlation, are presented in the equation below:

(1) $$Entropy=-\sum_{i=1}^{N_{g}}p(i)\log_{2}(p(i)+\varepsilon )$$

*Entropy* measures the average amount of information required to encode the image values, where $N_{g}$ = number of non-zero bins and $p\left(i\right)$ = normalized first order histogram.

(2) $$Contrast=\sum_{i=1}^{N_{g}}\sum_{j=1}^{N_{g}}{\left(i-j\right)}^{2}p(i,j)$$

*Contrast* determines local intensity variation present in the image. A larger value correlates with greater disparity in intensity values among neighboring voxels.

(3) $$Uniformity=\sum_{i=1}^{N_{g}}p{\left(i\right)}^{2}$$

*Uniformity* measures the sum of squares of each intensity value. Greater uniformity implies greater homogeneity. *Correlation* defines the linear dependency of grey level values to their respective voxels in GLCM. The value for correlation is between 0 (uncorrelated) and 1 (perfectly correlated). The equation of correlation is shown below:

(4) $$Correlation=\frac{\sum_{i=1}^{N_{g}}\sum_{j=1}^{N_{g}}p(i,j)ij-\mu_{x}\mu_{y}}{\sigma_{x}(i)\sigma_{y}(j)}$$

where $\mu_{x}$, $\mu_{y}$ and $\sigma_{x}$, $\sigma_{y}$ are mean grey level intensity and standard deviation of *p_x_* and *p_y_,* respectively.

### 2.4. Statistical Analysis

**Figure 1:**
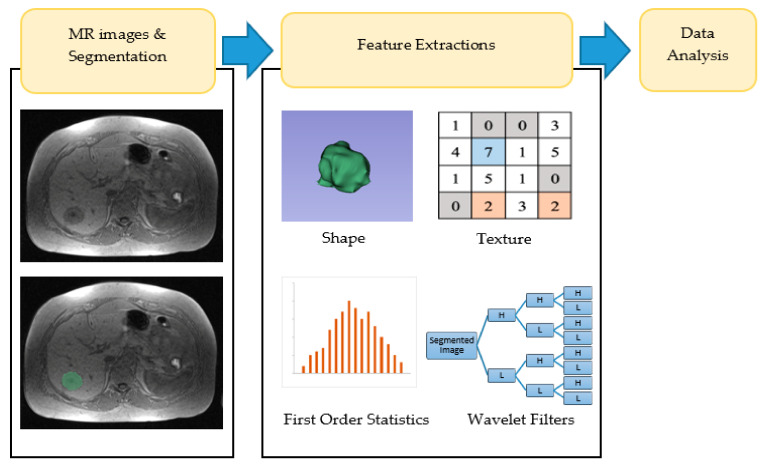
Schematic diagram of the analysis.

The intra-class correlation coefficient (ICC) is referred to as correlations within a class of data. It was calculated to quantify the reproducibility of features obtained. Figure 1 shows the flowcharts for reproducibility analysis in this study. ICC is a well-known statistical tool among researchers, ranging between 0 to 1. There are three type of ICC models which can be chosen appropriately according to experimental situation. For this study, variance estimates were obtained to determine the ICC for inter-observer segmentations by using a two-way mixed effect model of analysis of variance (ANOVA) [25]. Equation below defines the ICC:

(5) $$ICC(A,1)=\frac{MS_{R}-MS_{E}}{MS_{R}+(k+1)MS_{g}+\frac{k}{n}(MS_{C}-MS_{E})}$$

One-way analysis of variance (ANOVA) was used to obtained the *ICC* values for intra-observer segmentation [15,16]. Equation below defines *ICC* (*C*,1):

(6) $$ICC(C,1)=\frac{MS_{R}-MS_{W}}{MS_{R}+(k-1)MS_{W}}$$

where *MS_R_* = mean square for rows, *MS_W_* = mean square for residual sources of variance, *MS_E_* = mean square error, *MS_C_* = mean square for columns, *k* and n are number of observers involved and subjects. 

We evaluate intra-observers’ reproducibility by letting one observer segmentized two months apart of 30 patients and this demonstrates variation of data measured from two different sets of segmentation. Furthermore, this also helps to assess multiple initializations of segmentation algorithm from the same observer. For inter-observers’ reproducibility, the delineation was accomplished by several observers using the same methods of segmentation and the degree of agreement between different observers were analyzed. The difference of reproducibility for each segmentation were assessed using the Wilcoxon rank-sum test with *p*-value set at 0.05. All data were expressed in mean ± SD. All analyses of data were accomplished using Statistical Package for Social Sciences (SPSS, also known as IBM SPSS statistics) version 25 (SPSS Chicago, IL, USA).

## 3. Results

**Figure 2:**
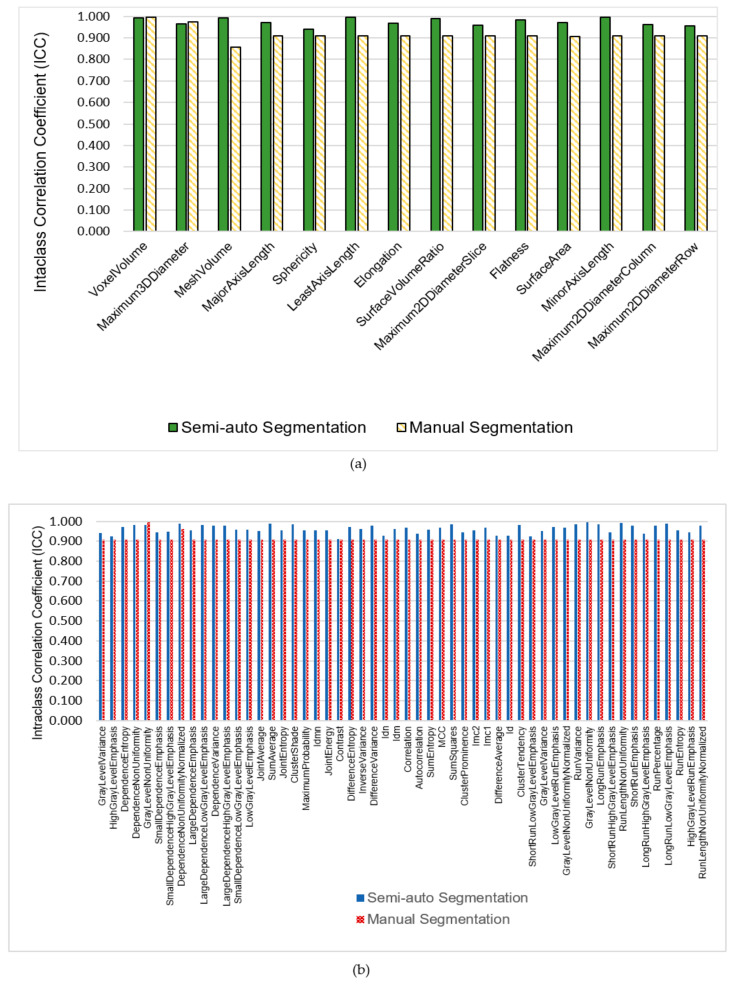

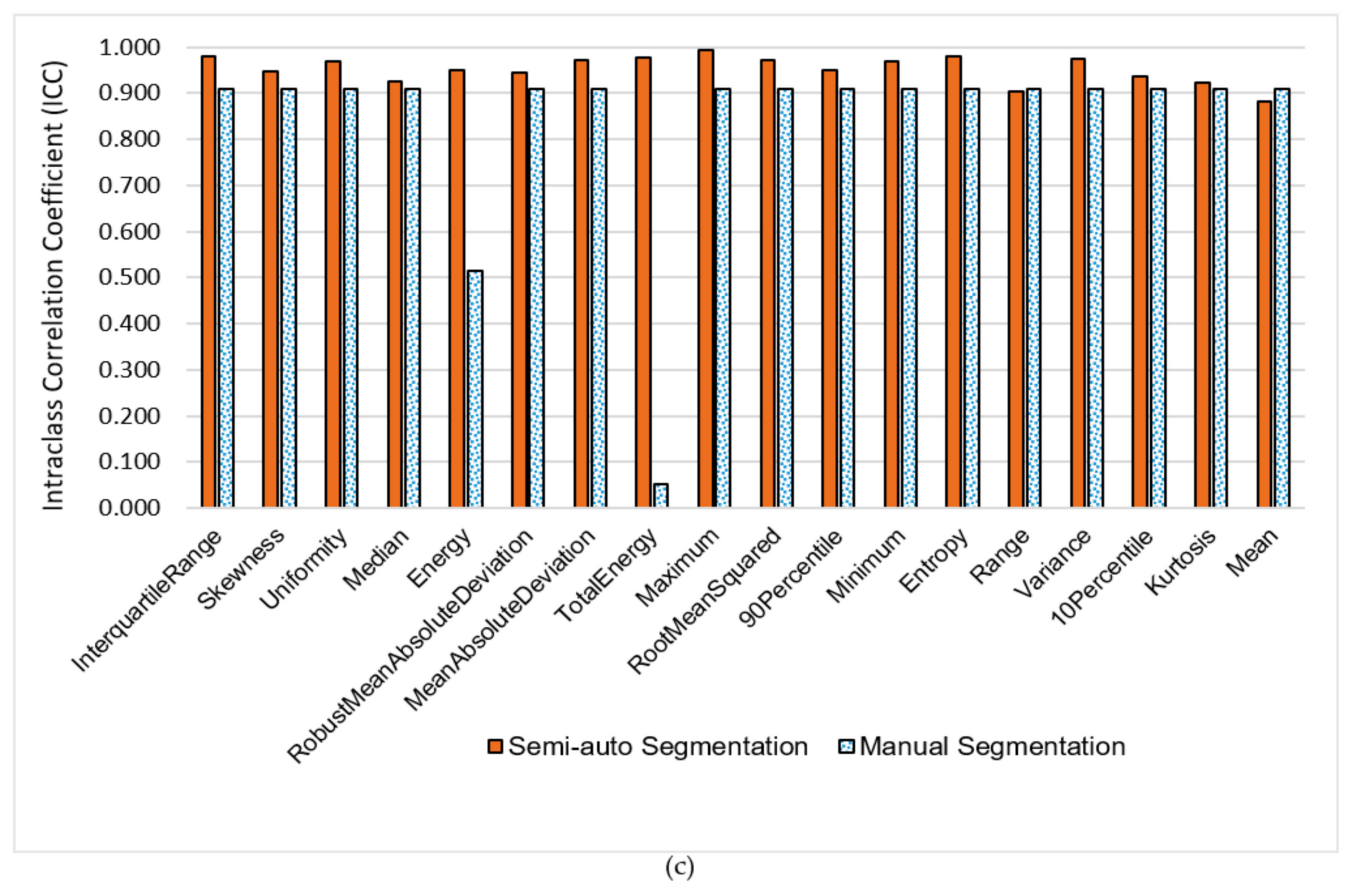
Comparisons of intra-class correlation coefficient (ICC) values in (**a**) shape based features, (**b**) textural features, and (**c**) first order statistics features.

In order to investigate the reliability of radiomic features extracted from 3D Slicer segmentation on MR images, a total of 662 features under four groups, (I) tumour intensity, (II) shape, (III) texture, and (IV) wavelet-features, were assessed. Of 30 subjects, the size of tumours for Stages III–IV (average size = 8.9 ± 3.4 cm) are larger than Stages I–II (average size = 3.7 ± 0.7 cm). We extracted radiomic from volume of interest determined by two independent observers twice using 3D Slicer software semi-automatic segmentation and compared them to manual segmentation by four independent observers. The semi-automatic segmentations were divided into two sets, each having two segmentations. Figure 2 shows comparisons of intra-class correlation coefficient between semi-automatic and manual segmentation in terms of categorized features. We observed that features extracted from semi-automatic segmentation had significantly higher reproducibility (average ICC = 0.952 ± 0.009, *p* < 0.05) compared to features extracted from manual segmentation (average ICC = 0.897 ± 0.011, *p* > 0.05).

**Figure 3:**
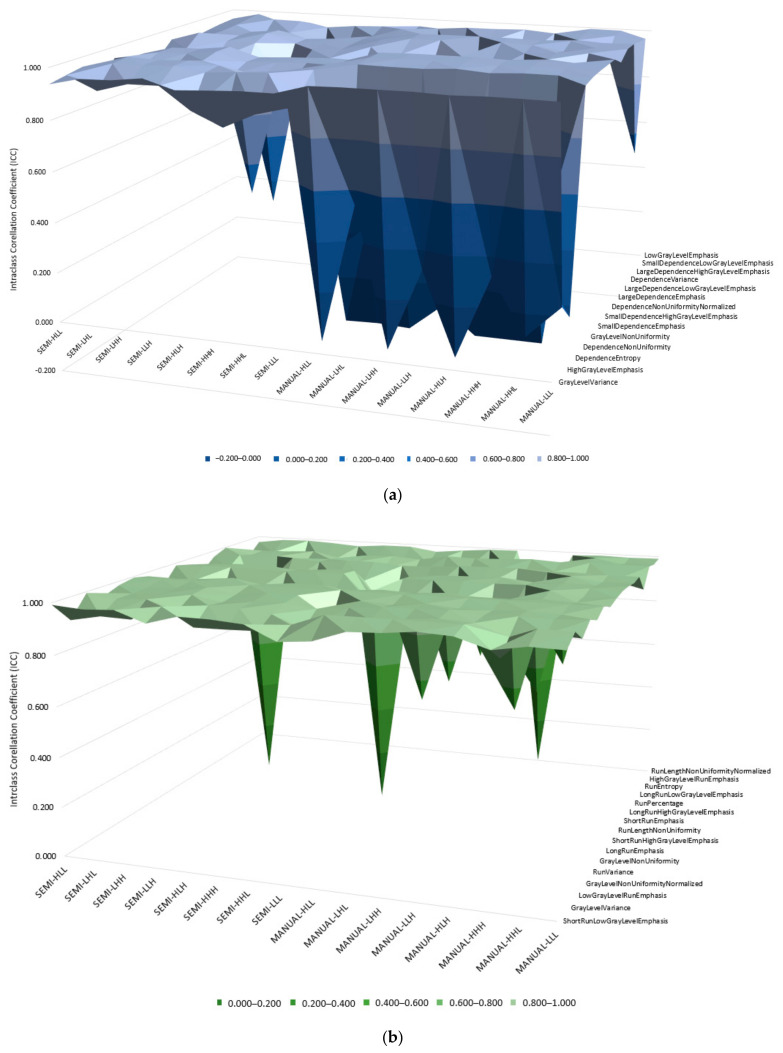
Comparisons of wavelet features for semi-automatic and manual segmentation in; (**a**) Gray-level dependence matrix (GLDM) features and, (**b**) first order statistics features.

There are 576 features of wavelet features, we separate the features into their respective feature types such as GLDM, GLCM, GLRLM and first order statistics for both semi-automatic and manual segmentations to ease the analysis process. There are 8 wavelet transforms in total. Figure 3 shows comparisons for ICC values of GLDM and first order features from both techniques. We observed that ICC values for intensity and texture features applied with wavelet transforms were high for semi-automatic segmentation compared to manual segmentation. Most ICC values for manual segmentations that undergoes wavelet transforms seen to have lower value which at lower level of the graphs. The ICC values of GLDM, GLCM, GLRLM, and intensity statistics for semi-automatic segmentation are 0.935 ± 0.008, 0.921 ± 0.013, 0.943 ± 0.011, and 0.948 ± 0.008, respectively. In contrary, ICC values for manual segmentations shows fluctuation in all four parameters (GLDM, GLCM, GLRLM, and intensity statistics) with average ICC equals to 0.796 ± 0.032, 0.848 ± 0.021, 0.941 ± 0.012, and 0.578 ± 0.037. Of the 662 features, 354 features (53%) showed higher ICC values for semi-automatic segmentation compared to manual one. Table 2, Table 3 and Table 4 show the reproducibility of ICC for both segmentations in regards with shape features and wavelet features.

Features that had been extracted are classified into three groups, high reproducibility (ICC ≥ 0.8), medium reproducibility (0.8 > ICC ≥ 0.5), and low reproducibility (ICC < 0.5), based on their ICC values. Out of 662 features, manual segmentation had 78.1% high, 6.5% medium, and 15.4% low reproducibility. However, semi-automatic segmentation, which is the flood filling effect, had 96.7% features in high reproducibility, 1.8% medium reproducibility, and 1.5% low reproducibility. Table 5 summarizes features extracted from both segmentations in reproducibility groups. Hence, reproducibility of the extracted features was higher for semi-automatic segmentations.

**Figure 4:**
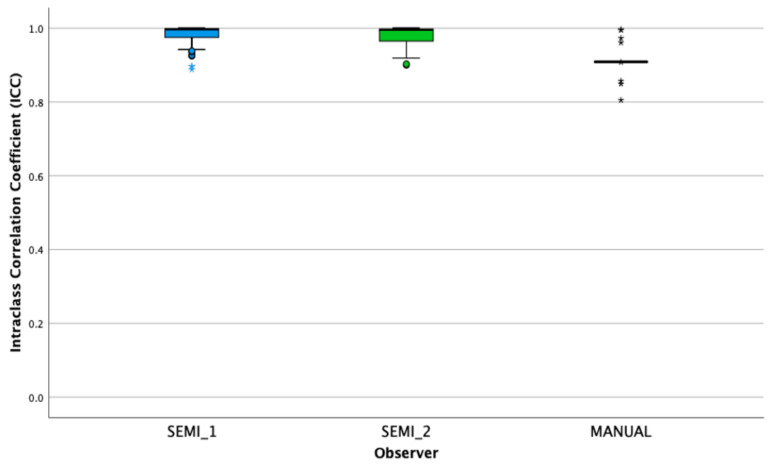
Boxplot of the ICC value represents by inter-observers’ reproducibility on the radiomic features.

The robustness of each technique was evaluated by analysing the ICC of features extracted from inter- and intra-observers. Based on Figure 4, high ICC values for inter-observer from semi-automatic segmentation were observed (ICC = 0.976 ± 0.006 and ICC = 0.978 ± 0.003, respectively). Table 6 tabulates the inter-observer reproducibility of shape features for one subject. 

## 4. Discussion

MR images were used in this study, as it produces high quality images compared to CT scans [34]. It is reported that resolution of input images influences the robustness of radiomic features as higher resolution images enhance visualization for segmentations [35]. In 2014, Chintan et al. assessed reproducibility of feature extracted using 3D-Slicer segmentation (GrowCut algorithm) which resulted in semi-automatic segmentation higher than manual delineation of CT images [7]. Therefore, additional features such as wavelet transform are implemented to study MR images using different types of algorithms. Despite increasing development in application of radiomic studies, robustness and reproducibility of radiomic features extracted from MR images are still one of the main challenges’ oncologist encounters.

In this study, we investigate the reproducibility and robustness of radiomic features of HCC between two types of segmentations, manual and semi-automatic, using 3D-Slicer software. A total of 662 features were extracted from volume of interest and can be classified into four main groups (18 tumour intensity, 14 shape features, 54 textural features, and 576 wavelet features). Based on the results, semi-automatic segmentation has higher ICC values compared to manual segmentation. Semi-automatic segmentation, which we used for flood filling algorithm, is also more robust and significantly higher compared to manual segmentation. Flood filling algorithm was performed when the observer chose several points around the volume of interest, and the tumour region was digitized according to the uniform colour which results in accurate segmentation [30]. This algorithm is proven to be the best segmentation method by comparing their accuracy in selection of objects [25].

All four quantitative imaging features extracted from semi-automatic segmentation had higher reproducibility score (*p* < 0.05) compared to manual segmentation. For most tumour intensity feature, semi-automatic segmentation had higher reproducibility compared to manual segmentation. There were a few negative ICC values for wavelet features in both segmentations shown in Figure 3, which indicates that particular features vary than any features randomly chosen from the whole population [36]. Despite that, the reproducibility of wavelet features from semi-automatic segmentation is still higher compared to manual segmentation. Furthermore, semi-automatic segmentation was consistent with ICC values (97% good reproducibility) compared to manual segmentation (78% good reproducibility). In 2020, Wang et al. implied that the features’ reliability increased using wavelet filters through their proposed deep learning tools compared to normal features [21].

We also analysed intra- and inter-observer reproducibility to evaluate the performance for both segmentations. Two sets from two different observers are shown in Figure 4 with one observer segments without formal clinical training. Despite using semi-automatic segmentation, experience of observers affects the segmentations of tumours as human interaction’s influence the process of segmentation. this indicates the difference in radiomic features. However, semi-automatic segmentation demonstrates high ICC values for both intra- and inter-observers compared to manual segmentation. This suggests that features extracted from semi-automatic segmentation are more robust and reproducible. There are two limitations in this study. First, this study does not involve image descriptors for prognostic models due to the insufficient images of HCC patients from the accessible source. Secondly, this study only comprises of single MRI sequence. The contrast enhanced MRI and infiltrative HCC are not included in this study.

## 5. Conclusions

This study assesses the reproducibility of radiomic features between two techniques. Semi-automatic segmentation using the flood fill algorithm produces more reproducible features, and this indicates it could be a better alternative to the current manual segmentation. Thus, this algorithm could be further applied for prognostic and classification models with sufficient data taken from several centers. Note that the study was focused on robustness and reproducibility of features extracted from semi-automatic and manual segmentation. Hence, for adapting machine learning, the study should be in larger scales with more data so the application will be much accurate.

## Figures and Tables

**Table 1:** Composition of 662 radiomic features extracted using 3D-Slicer.

Features	Original (n = 1)	Wavelet (n = 8)
Shape (n = 14)	14 × 1	-
Texture (n = 54)	54 × 1	54 × 8
Tumour Intensity (n = 18)	18 × 1	18 × 8

**Table 2:** Intra-class correlation coefficient (ICC) of semi-automatic and manual segmentation for shape and grey level run length matrix (GLRLM) features.

Features	Original	Semi-Automatic	Manual
Shape	Voxel Volume *	0.994	0.996
	Maximum 3D-Diameter	0.964	0.973
	Mesh Volume *	0.994	0.856
	Major Axis Length	0.972	0.909
	Sphericity	0.940	0.909
	Least Axis Length *	0.997	0.909
	Elongation	0.968	0.909
	Surface Volume Ratio *	0.990	0.909
	Maximum 2D-Diameter Slice	0.960	0.909
	Flatness	0.983	0.909
	Surface Area	0.971	0.908
	Minor Axis Length *	0.996	0.909
	Maximum 2D-Diameter Column	0.961	0.909
	Maximum 2D-Diameter Row	0.955	0.909
Gray-level run-length matrix (GLRLM)	Short Run Low Gray-Level Emphasis	0.925	0.909
	Gray-Level Variance	0.951	0.909
	Low Gray-Level Run Emphasis *	0.972	0.909
	Gray-Level Non-Uniformity Normalized	0.970	0.909
	Run Variance *	0.987	0.909
	Gray-Level Non-Uniformity *	0.996	0.909
	Long-Run Emphasis	0.985	0.909
	Short Run High Gray-Level Emphasis	0.946	0.909
	Run-Length Non-Uniformity *	0.991	0.909
	Short Run Emphasis	0.980	0.909
	Long Run High Gray-Level Emphasis	0.937	0.906
	Run Percentage *	0.978	0.909
	Long Run Low Gray-Level Emphasis *	0.989	0.907
	Run Entropy	0.954	0.909
	High Gray-Level Run-Emphasis	0.946	0.909
	Run-Length Non-Uniformity Normalized *	0.978	0.909
Tumour Intensity (First Order Statistics)	Interquartile Range *	0.980	0.909
	Skewness	0.947	0.909
	Uniformity	0.968	0.909
	Median	0.925	0.909
	Energy *	0.951	0.514
	Robust Mean Absolute Deviation	0.945	0.909
	Mean Absolute Deviation	0.971	0.909
	Total Energy *	0.978	0.053
	Maximum *	0.994	0.909
	Root Mean Squared	0.972	0.909
	90-Percentile	0.949	0.909
	Minimum	0.968	0.909
	Entropy	0.980	0.909
	Range	0.905	0.909
	Variance	0.975	0.909
	10-Percentile	0.937	0.909
	Kurtosis	0.922	0.909
	Mean	0.881	0.909

* Statistically significant (*p* < 0.05).

**Table 3:** Wavelet features for semi-automatic segmentation.

Features	Wavelet	Semi-Auto Segmentation							
		1.HLL	2.LHL	3.LHH	4.LLH	5.HLH	6.HHH	7.HHL	8.LLL
Gray-level run-length matrix (GLRLM)	Short Run Low Gray-Level Emphasis	0.992	0.983	0.989	0.947	0.981	0.979	0.970	0.915
	Gray-Level Variance	0.911	0.934	0.930	0.988	0.917	0.918	0.921	0.953
	Low Gray-Level Run Emphasis	0.995	0.979	0.986	0.949	0.978	0.974	0.961	0.915
	Gray-Level Non-Uniformity Normalized	0.960	0.980	0.986	0.990	0.968	0.979	0.982	0.967
	Run Variance	0.984	0.983	0.988	0.997	0.978	0.990	0.990	0.863
	Gray-Level Non Uniformity	0.996	0.997	0.996	0.996	0.995	0.995	0.996	0.997
	Long Run Emphasis	0.982	0.979	0.985	0.996	0.975	0.989	0.990	0.912
	Short Run High Gray-Level Emphasis	0.919	0.946	0.836	0.899	0.821	0.920	0.926	0.935
	Run-Length Non-Uniformity	0.990	0.995	0.996	0.994	0.992	0.996	0.997	0.883
	Short Run Emphasis	0.975	0.986	0.990	0.989	0.977	0.988	0.989	0.000
	Long Run High Gray-Level Emphasis	0.925	0.947	0.834	0.872	0.821	0.922	0.927	0.768
	Run Percentage	0.974	0.989	0.002	0.988	0.978	0.989	0.990	0.980
	Long Run Low Gray-Level Emphasis	0.998	0.971	0.971	0.936	0.971	0.966	0.943	0.908
	Run Entropy	0.951	0.973	0.976	0.983	0.958	0.977	0.980	0.971
	High Gray-Level Run Emphasis	0.920	0.947	0.836	0.896	0.820	0.921	0.927	0.935
	Run-Length Non-Uniformity Normalized	0.974	0.990	0.993	0.987	0.978	0.988	0.989	0.971
First Order Statistics	Interquartile Range	0.989	0.988	−0.001	0.991	0.975	0.926	0.990	0.989
	Skewness	0.945	0.968	0.966	0.986	0.945	0.950	0.956	0.935
	Uniformity	0.967	0.987	0.988	0.984	0.967	0.976	0.983	0.951
	Median	0.903	0.936	0.665	0.977	0.916	0.917	0.921	0.923
	Energy	0.912	0.934	0.929	0.988	0.916	0.917	0.921	0.953
	Robust Mean Absolute Deviation	0.919	0.946	0.836	0.869	0.820	0.921	0.927	0.935
	Mean Absolute Deviation	0.949	0.961	0.970	0.981	0.945	0.974	0.973	0.973
	Total Energy	0.994	0.993	0.996	0.993	0.994	0.995	0.997	0.996
	Maximum	0.994	0.994	0.994	0.994	0.994	0.994	0.994	0.994
	Root Mean Squared	0.976	0.986	0.983	0.984	0.978	0.969	0.976	0.974
	90-Percentile	0.910	0.923	0.833	0.952	0.828	0.906	0.912	0.940
	Minimum	0.976	0.983	0.970	0.982	0.975	0.933	0.963	0.965
	Entropy	0.977	0.987	0.992	0.991	0.978	0.991	0.991	0.933
	Range	0.999	0.976	0.977	0.965	0.970	0.965	0.942	0.845
	Variance	0.989	0.996	0.987	0.986	0.993	0.953	0.975	0.867
	10-Percentile	0.954	0.909	0.858	0.770	0.987	0.898	0.913	0.949
	Kurtosis	0.971	0.964	0.956	0.963	0.994	0.988	0.991	0.946
	Mean	0.995	0.980	0.986	0.954	0.997	0.974	0.961	0.922

**Table 4:** Wavelet features for manual segmentation.

Features	Wavelet	Manual Segmentation							
		1.HLL	2.LHL	3.LHH	4.LLH	5.HLH	6.HHH	7.HHL	8.LLL
Gray-level run-length matrix (GLRLM)	Short Run Low Gray-Level Emphasis	0.926	0.969	0.974	0.987	0.980	0.942	0.963	0.960
	Gray-Level Variance	0.998	0.999	0.999	0.999	0.998	0.887	1.000	0.998
	Low Gray-Level Run Emphasis	0.924	0.965	0.972	0.987	0.979	0.938	0.957	0.950
	Gray-Level Non-Uniformity Normalized	0.980	0.991	0.993	0.997	0.983	0.989	0.986	0.993
	Run Variance	0.984	0.976	0.989	0.978	0.980	0.987	0.981	0.968
	Gray-Level Non Uniformity	0.996	0.996	0.996	0.997	0.996	0.996	0.996	0.998
	Long Run Emphasis	0.985	0.979	0.988	0.979	0.979	0.987	0.981	0.972
	Short Run High Gray-Level Emphasis	0.872	0.516	0.927	0.716	0.506	0.999	0.980	0.984
	Run-Length Non-Uniformity	0.990	0.991	0.995	0.991	0.993	0.996	0.994	0.991
	Short Run Emphasis	0.983	0.990	0.994	0.996	0.984	0.990	0.986	0.989
	Long Run High Gray-Level Emphasis	0.869	0.507	0.925	0.675	0.490	0.996	0.979	0.981
	Run Percentage	0.982	0.992	0.995	0.996	0.986	0.991	0.988	0.988
	Long Run Low Gray-Level Emphasis	0.915	0.939	0.962	0.955	0.969	0.938	0.945	0.914
	Run Entropy	0.992	0.989	0.993	0.999	0.992	0.996	0.995	0.994
	High Gray-Level Run Emphasis	0.872	0.515	0.927	0.708	0.504	0.999	0.980	0.984
	Run-Length Non-Uniformity Normalized	0.983	0.993	0.995	0.001	0.987	0.992	0.989	0.991
First Order Statistics	Interquartile Range	−0.005	0.021	0.087	0.033	0.010	0.026	−0.007	−0.008
	Skewness	0.994	0.998	0.998	0.999	0.995	0.999	0.999	0.999
	Uniformity	0.972	0.985	0.988	0.992	0.982	0.984	0.984	0.957
	Median	0.998	0.999	0.999	0.999	0.999	1.000	1.000	0.997
	Energy	0.000	0.000	0.000	0.000	0.000	0.000	0.000	0.000
	Robust Mean Absolute Deviation	0.872	0.515	0.927	0.708	0.504	0.999	0.980	0.984
	Mean Absolute Deviation	0.096	0.946	0.958	0.918	0.867	0.971	0.925	0.057
	Total Energy	0.000	0.001	0.030	0.000	0.002	0.051	0.002	0.000
	Maximum	0.996	0.996	0.996	0.997	0.996	0.996	0.996	0.997
	Root Mean Squared	−0.018	0.034	0.315	0.010	−0.007	0.242	0.031	−0.007
	90-Percentile	0.873	0.517	0.928	0.853	0.504	0.999	0.980	0.990
	Minimum	0.001	0.006	0.007	0.013	0.000	0.004	−0.001	−0.025
	Entropy	0.977	0.972	0.976	0.972	0.970	0.964	0.965	0.949
	Range	0.548	0.953	0.979	0.787	0.965	0.927	0.939	0.183
	Variance	0.102	0.912	0.981	0.837	0.560	0.974	0.630	0.065
	10-Percentile	0.888	0.718	0.890	0.600	0.587	0.964	0.969	0.921
	Kurtosis	−0.005	−0.006	−0.007	−0.002	−0.006	−0.005	−0.010	0.000
	Mean	0.918	0.961	0.981	0.423	0.978	0.930	0.957	0.002

**Table 5:** Features extracted in different reproducibility groups.

Reproducibility Groups	Semi-Automatic	Manual
High (ICC ≥ 0.8)	639 (96.7%)	517 (78.1%)
Medium (0.8 > ICC ≥ 0.5)	12 (1.8%)	43 (6.5%)
Low (ICC < 0.5)	11 (1.5%)	102 (15.4%)

**Table 6:** ICC of radiomic features.

Features		Semi_1	Semi_2	Manual
Shape	Voxel Volume *	0.984	0.996	0.996
	Maximum 3D-Diameter	0.878	0.965	0.973
	Mesh Volume	0.985	0.996	0.857
	Major Axis Length	0.939	0.956	0.909
	Sphericity	0.756	0.903	0.909
	Least Axis Length	0.991	0.999	0.909
	Elongation *	0.941	0.932	0.909
	Surface Volume Ratio	0.962	0.977	0.909
	Maximum 2D-Diameter Slice	0.924	0.944	0.909
	Flatness	0.970	0.954	0.909
	Surface Area	0.941	0.988	0.908
	Minor Axis Length	0.984	0.998	0.909
	Maximum 2D-Diameter Column	0.886	0.995	0.909
	Maximum 2D-Diameter Row	0.866	0.965	0.909
Gray-level run-length matrix (GLRLM)	Short Run Low Gray-Level Emphasis *	0.964	0.927	0.909
	Gray-Level Variance	0.999	0.998	0.909
	Low Gray-Level Run Emphasis	0.983	0.962	0.909
	Gray-Level Non-Uniformity Normalized	0.997	0.998	0.909
	Run Variance	0.993	0.999	0.909
	Gray-Level Non-Uniformity	0.995	0.995	0.909
	Long-Run Emphasis	0.992	0.999	0.909
	Short Run High Gray-Level Emphasis	0.943	0.927	0.909
	Run-Length Non-Uniformity	0.996	0.998	0.909
	Short Run Emphasis	0.999	0.999	0.909
	Long Run High Gray-Level Emphasis *	0.937	0.922	0.909
	Run Percentage	0.999	0.999	0.909
	Long Run Low Gray-Level Emphasis	0.986	0.998	0.909
	Run Entropy	0.997	0.998	0.909
	High Gray-Level Run-Emphasis *	0.943	0.927	0.909
	Run-Length Non-Uniformity Normalized	0.999	0.999	0.909
Tumour Intensity (First Order Statistics)	Interquartile Range	0.98	0.989	0.909
	Skewness	0.999	0.996	0.909
	Uniformity	1	0.994	0.909
	Median	1	0.993	0.909
	Energy	0.999	0.998	0.805
	Robust Mean Absolute Deviation	0.942	0.926	0.909
	Mean Absolute Deviation	0.991	0.998	0.909
	Total Energy	1	0.992	0.849
	Maximum	0.999	0.992	0.909
	Root Mean Squared	0.997	0.995	0.909
	90-Percentile *	0.945	0.919	0.909
	Minimum	0.995	0.987	0.909
	Entropy	0.999	0.999	0.909
	Range	0.998	0.998	0.909
	Variance	0.999	0.997	0.909
	10-Percentile	0.944	0.928	0.909
	Kurtosis	0.888	0.9	0.909
	Mean	0.988	0.975	0.909

* Statistically significant (*p* < 0.05).
